# Topology-guided polar ordering of collective cell migration

**DOI:** 10.1126/sciadv.adk4825

**Published:** 2024-04-17

**Authors:** Emma Lång, Anna Lång, Pernille Blicher, Torbjørn Rognes, Paul Gunnar Dommersnes, Stig Ove Bøe

**Affiliations:** ^1^ Department of Microbiology, Oslo University Hospital, Oslo, Norway.; ^2^ Department of Medical Biochemistry, Institute of Clinical Medicine, University of Oslo, Oslo, Norway.; ^3^ Centre for Bioinformatics, Department of Informatics, University of Oslo, Oslo, Norway.; ^4^ Department of Physics, Norwegian University of Science and Technology, Trondheim, Norway.

## Abstract

The ability of epithelial monolayers to self-organize into a dynamic polarized state, where cells migrate in a uniform direction, is essential for tissue regeneration, development, and tumor progression. However, the mechanisms governing long-range polar ordering of motility direction in biological tissues remain unclear. Here, we investigate the self-organizing behavior of quiescent epithelial monolayers that transit to a dynamic state with long-range polar order upon growth factor exposure. We demonstrate that the heightened self-propelled activity of monolayer cells leads to formation of vortex-antivortex pairs that undergo sequential annihilation, ultimately driving the spread of long-range polar order throughout the system. A computational model, which treats the monolayer as an active elastic solid, accurately replicates this behavior, and weakening of cell-to-cell interactions impedes vortex-antivortex annihilation and polar ordering. Our findings uncover a mechanism in epithelia, where elastic solid material characteristics, activated self-propulsion, and topology-mediated guidance converge to fuel a highly efficient polar self-ordering activity.

## Teaser

Proliferation and annihilation of vortex-antivortex pairs orchestrate collective cell migration in epithelia.

## INTRODUCTION

Collective cell migration refers to the coordinated movement of groups of cells, which is essential for a variety of biological processes, including embryonic development, wound healing, and cancer metastasis. To achieve such coherent motion, cells rely on a range of biophysical mechanisms to communicate with each other and their environment (*1*, *2*). However, during the past decade, it has become increasingly clear that biological mechanisms alone do not govern the dynamic behavior of cells and tissues. Instead, the behavior of cell collectives is dictated by the interplay between biological functions and active matter physics, a branch of physics that deals with self-propelled entities and their capacity to generate nonequilibrium phenomena such as complex collective behaviors (*3*, *4*).

A prominent example of active matter in biology comes from studies demonstrating the presence of active nematic order, a class of active matter that involves the alignment of particles with a bipolar orientation. Nematic order has been observed in various biological systems, ranging from intracellular structures to long-range tissue organization (*5*). Consequently, active topological defects, typically associated with nematic order and characterized by charges of +1/2 and −1/2, have been shown to influence several biological functions such as tissue organization (*6*–*12*), extrusion of apoptotic cells (*13*), microtubule dynamics (*14*–*16*), biofilm formation (*17*), and cancer cell spreading (*18*). However, dynamic biological processes that involve collective unidirectional migration of large groups of cells may be explained by active polar ordering, which represents a class of active matter where each particle has self-propulsion in a distinct polar orientation. Within active polar materials, a prevalent outcome is the emergence of dynamic flow fields embedded with vortices and antivortices. These are defined by having +1 and −1 topological charges, respectively. Notably, our understanding of the role played by vortices and antivortices, also referred to as +1 and −1 defects, in dynamic polarized biological tissues remains limited.

Although the presence of ±1 defects in polar systems is associated with disruption of uniform polar ordering, pairs of oppositely charged defects can also exert mutual attraction and eliminate each other through annihilation, resulting in increased polar order. In static systems such as ferromagnetic fields, this process can lead to the elimination of domain walls and improved magnetic field structure (*19*, *20*). Furthermore, recent studies have demonstrated the power of ±1 defect pair annihilation in polar self-ordering in a field of spinning colloid particles in confinement. In this particular system, the activation of particle self-propulsion triggers the emergence of both +1 and −1 defects, which subsequently facilitate the establishment of pristine polar alignment by mutually annihilating defects of opposite charges (*21*, *22*). Despite the demonstrated efficacy of this fundamental physical mechanism in generating long-range polar order within a self-propelled particle field, it remains uncertain whether biological tissues can exploit the same process to facilitate long-range coordination of migrating cell collectives.

Here, we explore motility-induced self-organization of polarity alignment within keratinocyte monolayers that have been stimulated to exit from an immobile quiescent state (*23*, *24*). We uncover the presence of multiple ±1 defect pairs at early stages after activation of cell migration, which, due to their positional order and spin orientation, actively steer the ordering process through mutual attraction and subsequent annihilation of oppositely charged defect pairs. By manipulating the intercellular affinity, we demonstrate that effective annihilation-mediated velocity alignment is correlated with the ability of the monolayer to maintain stable interactions between neighboring cells during the self-ordering process. In addition, we faithfully replicate our experimental observations using a minimal computational model treating the monolayer as an active elastic solid (AES). Last, we demonstrate how the underlying principles of topology-mediated guidance actively contribute to a process of polar flipping, effectively reversing the orientation of polar motility over large distances. Our study demonstrates how motility-activated local-scale vortex-antivortex pairs give rise to large-scale polar order of the velocity direction in AESs, such as epithelial monolayers.

## RESULTS

### Vortex-antivortex pair annihilation mediates self-ordering of collective cell migration

**Fig. 1.**
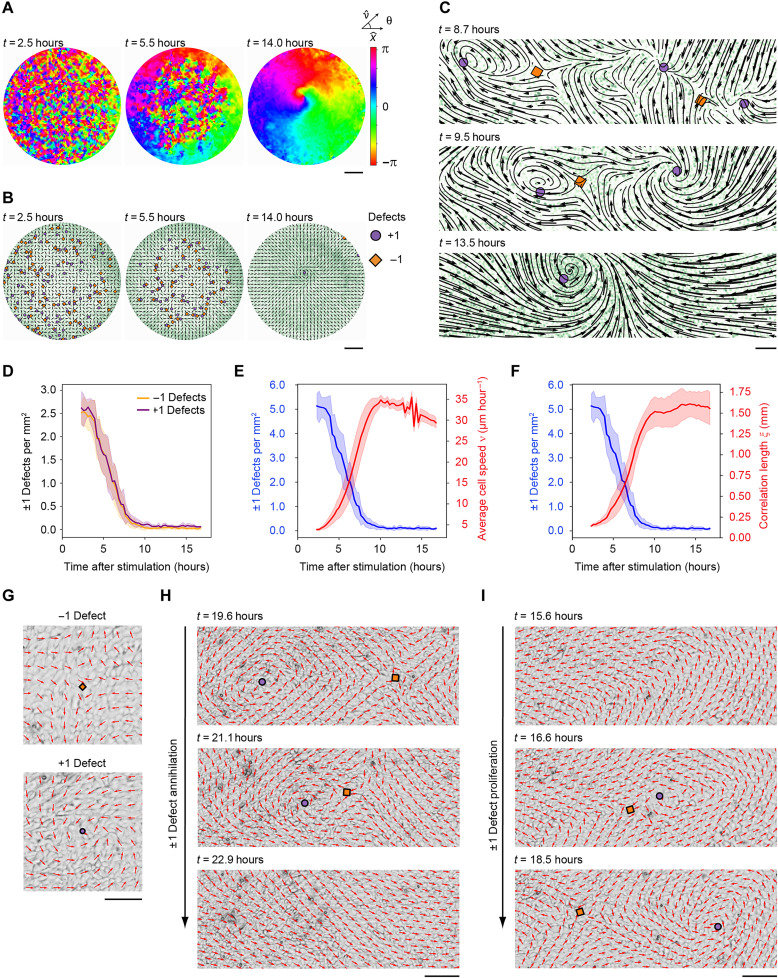
Vortex-antivortex pair annihilation mediates self-ordering of collective cell migration. (**A** to **F**) HaCaT cells were seeded in 96-well plates before serum deprivation and subsequent serum activation at *t* = 0. (A) Time evolution of polar ordering. The color map represents the angle of the local velocity field relative to the *x* axis. The surface of a whole circular monolayer is shown. Scale bar, 1 mm. (B) Detection and mapping of ±1 defects within the emergent velocity field. Nuclei are labeled with mCherry-tagged histone H2B (green), and the normalized vector field shows the direction of cell migration. Circles and diamonds indicate +1 and −1 defects, respectively. Data have been extracted from the same monolayer as in (A). Scale bar, 1 mm. See also movie S1. (C) Zoom-in on the final ordering steps leading to the formation of a single stable +1 defect. Streamline plots generated by particle image velocimetry (PIV) and mCherry-tagged histone H2B (green) are shown. The ±1 defects are denoted as in (B). Scale bar, 100 μm. (D to F) Time evolution of −1 and + 1 defect densities (D), ±1 defect densities (blue line) and the average cell migration speed ν (red line) (E), and ±1 defect densities (blue line) and the spatial correlation length ξ (red line) (F) after serum activation at *t* = 0. (D to F) Graphs represent average values ± SD from *n* = 8 separate monolayers. (**G** to **I**) HaCaT cells were seeded in 12-well plates before serum deprivation and subsequent serum activation. The images show the normalized velocity field (red arrows) superimposed on phase contrast images. The ±1 defects are denoted as in (B). (G) Snapshots showing examples of stable −1 (top) and +1 (bottom) defects. Scale bar, 100 μm. See also movies S2 and S3. (H) Time series showing annihilation of a ± 1 defect pair. Scale bar, 100 μm. See also movie S4. (I) Time series showing spontaneous formation of a ±1 defect pair. Scale bar, 100 μm. See also movie S5.

The experimental approach used in this study to investigate long-range polar ordering of migrating cells within two-dimensional (2D) keratinocyte monolayers is based on a previously described model (*23*, *24*). The method involves using quiescent HaCaT keratinocyte collectives that are confined to circular surfaces of 96-well glass bottom plates with a well diameter of 7 mm. Cells are initially induced into a static quiescent state by 3 days of serum depletion. Subsequently, cells are released from this quiescent state through serum exposure, which initiate a migratory behavior leading to a coordinated pattern of movement toward the center of the well. This transition from disorder to system-spanning order of the velocity direction occurs within 10 hours after serum stimulation, highlighting the presence of a powerful and effective self-organizing mechanism (Fig. 1A) (*23*). Notably, the dynamic behavior observed for motility-induced HaCaT cells on round surfaces bears notable similarities to that observed in activated colloid particles, wherein the activation of particle self-propulsion and annihilation of vortex-antivortex pairs drives a coarsening process, ultimately leading to macroscopic vortices with a single +1 defect at the center (*21*, *22*, *25*).

To investigate whether the self-organization of serum-activated quiescent keratinocyte monolayers is associated with an integer defect-mediated coarsening dynamics similar to that observed for colloid particles, we monitored ±1 defects in HaCaT keratinocyte monolayers as cells underwent motility-induced self-ordering after exposure to fetal bovine serum (FBS). We identified +1 and −1 defects by calculating the local winding number for each grid point of the velocity field using a computer-based algorithm. We detected multiple defects in the early phase of self-ordering, which decreased progressively as polar order increased over time (Fig. 1, B and C, and movie S1). The ordering process consistently propagated from the edges to the center of the circular domain, with a single +1 defect (with an equal probability of having either a clockwise or a counterclockwise spin orientation) persisting at the center of the monolayer once maximal order had been reached (Fig. 1, A to C). We detected a nearly equal number of +1 and −1 defects, indicating a balance between the topological charges throughout the ordering process (Fig. 1D). Moreover, the number of ±1 defects present in the monolayers was inversely correlated with the average cell migration speed ν (Fig. 1E) and the spatial correlation length ξ (Fig. 1F).

During the serum-induced ordering phase in 96-well plates, the high density and short lifetime of ±1 defects made it challenging to capture the dynamic behavior of individual defects of both charges over time. To overcome this challenge, we expanded the cell monolayer dimension to a diameter of 35 mm using 12-well plates. The larger surface area prevented the system from achieving full topological order and greatly increased the probability of capturing integer defects of both charges that were stable for several hours at low defect densities (Fig. 1G and movies S2 and S3). Temporal analysis of defects under these conditions revealed mutual attraction between +1 and −1 defects, resulting in annihilation and instantaneous polar ordering in the velocity field (Fig. 1H and movie S4). In rare instances, we also observed defect proliferation, where a vortex-antivortex pair spontaneously formed and subsequently moved apart in opposite directions (Fig. 1I and movie S5). The local motility patterns formed by defect pairs before and after annihilation are similar to those previously observed in non-biological systems such as magnetic fields and active colloids (*21*, *26*). For example, the directed migration between a ±1 defect pair invariably undergoes a 180° reversal following defect annihilation (Fig. 1H and movie S4).

Unlike colloid particles, which have a uniform shape, HaCaT cells in a monolayer can vary the shape of their perimeter. To investigate whether cells have a preference for migrating along their length axis, we calculated a shape tensor field across the monolayer and compared it to the velocity direction at each grid point (*9*). Calculation of the angles between the tensor field and the velocity field revealed a largely independent relationship between velocity direction and cell shape over time (fig. S1).

We also examined whether the observed migration pattern is influenced by the circular geometry of the confinement by using 96-well glass bottom plates with squared instead of circular wells. The rate of ±1 defect annihilation and self-ordering observed in a squared shaped geometry was comparable to that observed in a circular confinement (fig. S2 and movie S6). However, on squared surfaces, the final motility pattern was less uniform, and the system rarely reached a single-defect state, suggesting that the geometry influences the motility pattern in this system.

Last, we examined the dynamics of motility-induced self-ordering in a scenario where cells can move into an open area. For these experiments, monolayers in 12-well plates were initially programmed into quiescence by serum deprivation and then “scratched” to create a wound in the monolayer before stimulating cell motility by serum activation. We observed effective coarsening and orientation of the velocity field in the direction of the open wound concomitant with defect elimination. Furthermore, annihilation of vortex-antivortex pairs was found to facilitate the orientation of collective cell migration in the direction of the wound more than 2 mm beyond the wound edge (fig. S3 and movie S7).

In summary, the motility-induced polar ordering of serum-activated HaCaT keratinocyte monolayers is dictated by ±1 defects and exhibits notable parallels to a topology-driven coarsening process previously described for active polar fluids (*21*, *22*, *25*).

### An AES model recapitulates annihilation-mediated ordering

To model the behavior of serum-stimulated quiescent cell sheets using numerical simulation, we used a minimal model that considers the intrinsic elasticity of epithelial monolayers as well as the stable interactions between neighboring cells. We also considered the fact that the cells develop dynamic polar order (both locally and globally), which suggests the presence of orientational interactions between neighboring cells. The model does not incorporate considerations of cell shape or the ability of cells to coordinate their migratory direction with their shape orientation because our observations indicate that the orientation of cell shapes and the direction of cell migration are independent parameters in migrating collectives of HaCaT cells (fig. S1). To capture these dynamics, we used an AES model

$${\dot{R}}_{i}=V_{c}\;P_{i}+\frac{1}{\zeta }F_{i}$$ (1)

$${\dot{P}}_{i}=\gamma \;\left(P_{i}\times {\dot{R}}_{i}\right)\times P_{i}$$ (2)

where ***R****_i_* is the cell positions and ***P****_i_* the polarity of cell propulsion (direction of propulsion), *V_c_* is the cell propulsion speed in the absence of elastic force, ***F****_i_* the elastic force on the cell, and ζ the substrate friction. While Eq. 1 can be considered to express balance of forces, Eq. 2 gives the polar ordering effect and implies that the direction of propulsion is turning toward the direction of the velocity with a rate constant γ. In the original AES model, the particles are arranged in a hexagonal bead-spring lattice, with linear elastic springs giving forces ***F****_i_* on each particle (*27*). However, because long-range hexagonal order is not observed in HaCaT monolayers, we used previously described implementations of the model that represent the cell monolayer as a bead-spring network with springs of different lengths (fig. S4A) (*28*–*32*). This approach ensures isotropic elasticity globally. In simulations, all particles were given an initial random orientation and a maximal self-propulsion speed of 35 μm hour^−1^.

**Fig. 2.**
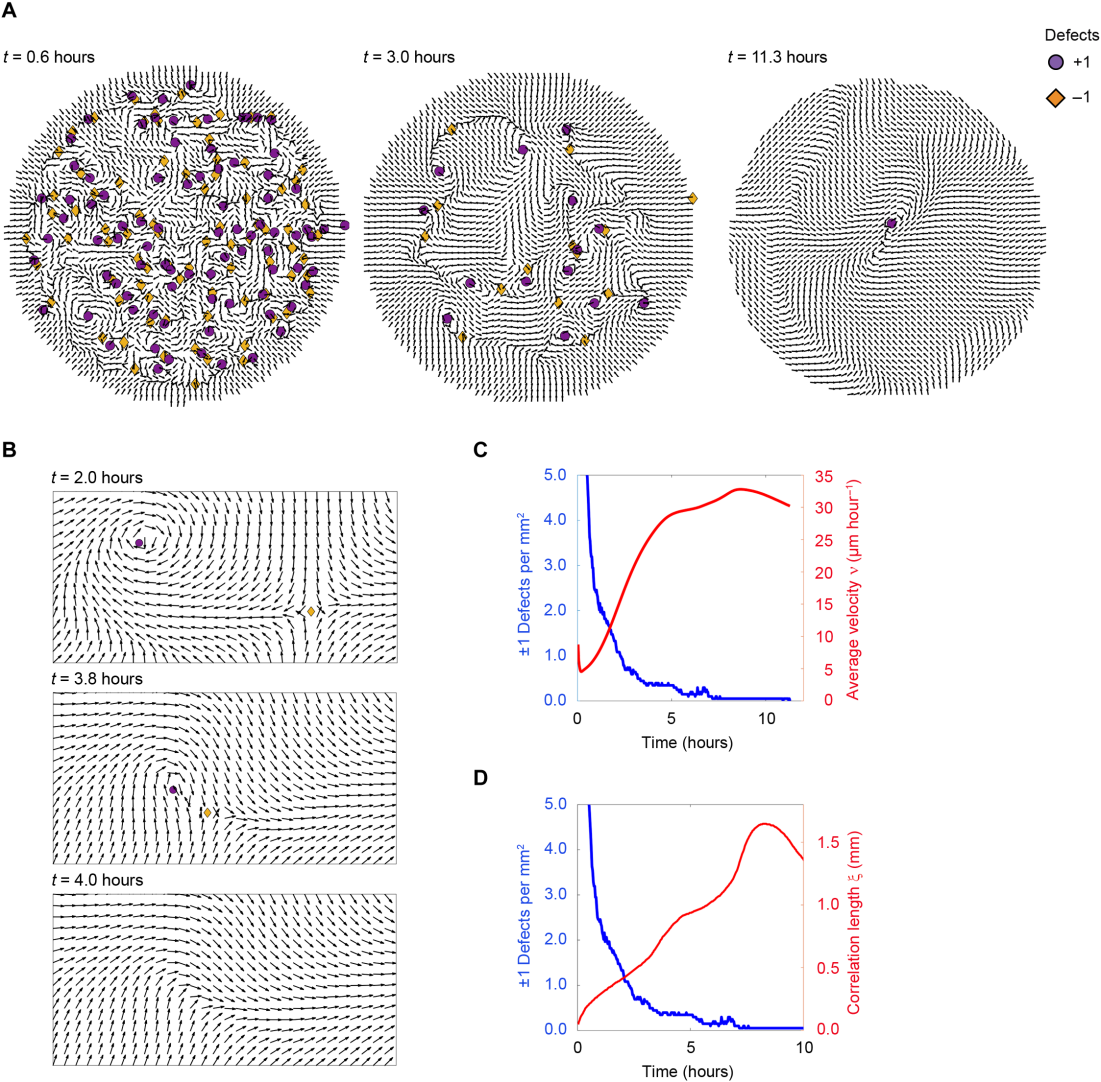
An AES model recapitulates annihilation-mediated ordering. (**A**) Numerical simulation showing defect annihilation and coarsening of the velocity direction field over time. Circles and diamonds indicate computer-based detection of defects with charge +1 and −1, respectively. The dimensionless rigidity and turnings rate are $\tilde{K}=40$ and $\tilde{\gamma }=1.5$ . The field contains *N* =100,000 particles. See also movie S8. (**B**) Zoomed-in snapshots from numerical simulations showing mutual attraction and annihilation of a defect pair. Arrows represent the normalized velocity field. Circles and diamonds indicate computer-based detection of +1 and −1 defect cores, respectively. (**C**) Time evolution of ±1 defect densities (blue line) and average velocity ν (normalized by cell velocity 35 μm hour^−1^, red line) during the self-ordering process in numerical simulations. (**D**) Time evolution of ±1 defect densities (blue line) and the correlation length ξ (red line) during the self-ordering process in numerical simulations.

Despite its minimal nature, we found that the model reproduces many of the key experimental findings. First, the model shows that the emergence of long-range order in the velocity field initially develops locally in the form of oppositely charged pairs of vortices and antivortices, which subsequently attract and annihilate each other (Fig. 2, A and B, and fig. S4, B to D, and movie S8). This process continues until macroscopic order is achieved, with typically one +1 defect remaining in the center of the system (Fig. 2, A and B, and movie S8). Moreover, the numerical simulations also recapitulate the anticorrelation between defect density and speed, as well as the anticorrelation between defect density and correlation length, which develops over time (Fig. 2, C and D). Therefore, a model with isotropic elasticity and the assumption of “turning with the velocity direction” is sufficient to replicate the topology-guided coarsening dynamics that we observe in our experiments.

### Vortex-antivortex pair annihilation and polar self-ordering are affected by intercellular stability

Previous studies have indicated that reactivation of quiescent HaCaT cell sheets relies on epidermal growth factor (EGF) receptor activity and that quiescence-dependent movement can be induced by EGF alone, without the presence of FBS (*24*). By analyzing ±1 defect dynamics in FBS-treated versus EGF-treated quiescent monolayers, we observed a slower rate of defect elimination in monolayers reactivated with EGF (fig. S5A). In addition, FBS-stimulated cells exhibited higher and more rapidly increasing spatial correlation lengths ξ than cells stimulated with EGF alone (fig. S5B). To evaluate the relative dynamics among cells in the monolayer, we used an assay that quantifies the length of cell trajectories in relation to a selected anchor cell that we computationally maintain at a stationary reference point (as depicted in fig. S5C). This measurement, which we refer to as relative motility, indicates the positional stability of cells in the monolayer relative to each other. The analysis showed that EGF-treated cells exhibit an increase in relative motility compared to cells in FBS-treated monolayers (fig. S5, D and E). These data suggest that defect-mediated polar coarsening may be affected by the ability of the monolayer to maintain stable connections between neighboring cells.

**Fig. 3.**
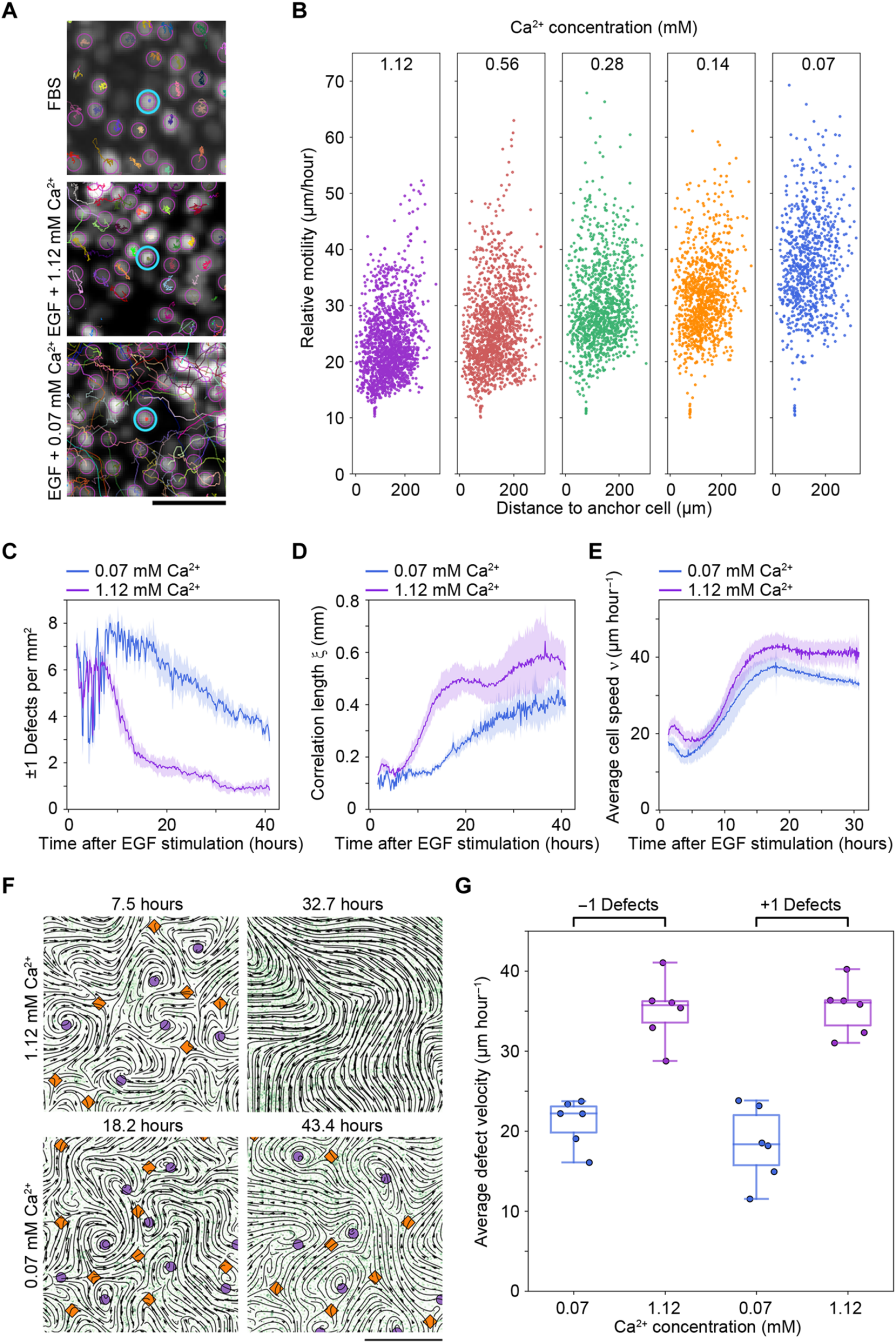
Defect annihilation and polar self-ordering are affected by intercellular stability. (**A** and **B**) Intercellular plasticity was monitored by calculating the relative motility of cells in relation to an anchor cell. (A) Fluorescence microscopy images showing mCherry-labeled nuclei after stimulation with FBS (top), EGF in the presence of high calcium concentration ([Ca^2+^] = 1.12 mM; middle), or low calcium concentration ([Ca^2+^] = 0.07 mM; bottom). Cell trajectories, which represent the relative motility, are shown in relation to a reference anchor cell (highlighted by a blue circle). Scale bar, 50 μm. See also movie S9. (B) Scatter plots depicting the relative motility (micrometers per hour) of cells relative to an anchor cell at different calcium concentrations. Analysis was performed on *n* = 12 field of view per sample. See also (A) and fig. S5C. (**C** to **E**) Time evolution of ±1 defects (C), the spatial correlation length ξ (D), and the average cell migration speed ν (E) is shown for monolayers exposed to EGF and low or high calcium concentrations at time *t* = 0. Average values ± SD are presented for *n* = 8 separate monolayers. Figure S5 (G to I) provides a complete range of the different Ca^2+^ concentrations tested. (**F**) Computer-based detection of +1 (purple circles) and −1 (orange diamonds) defects following EGF stimulation in the presence of high or low calcium concentration. The direction of cell migration is demonstrated by streamline plots (black lines). The mCherry-tagged cell nuclei are colored in green. Scale bar, 1000 μm. See also movies S10 and S11. (**G**) Boxplot depicting the average velocity for −1 and +1 defects in monolayers treated with EGF under conditions with high or low calcium concentration. Average values are shown for *n* = 6 separate monolayers per sample. The whiskers include minimum and maximum values.

To examine the potential impact of relative cell movement on ±1 defect annihilation and polar self-ordering in more detail, we varied the calcium concentration ([Ca^2+^]) in the cell culture medium. A low [Ca^2+^] is known to cause reduced stability of neighbor cell interactions in epithelial tissues, while a high concentration leads to increased stability (*24*). In agreement with this, lowering the [Ca^2+^] in our experiments resulted in increased relative motility, while increasing [Ca^2+^] showed the opposite effect (Fig. 3, A and B, fig. S5F, and movie S9). In addition, by analyzing the changes in average squared distance between pairs of migrating cells over time, which captures their ability to diffuse from each other, we observed a solid-to-liquid phase transition when going from high to low [Ca^2+^] (fig. S5, J and K, and table S1). By analyzing ±1 defect densities and the spatial correlation length ξ after EGF stimulation at different [Ca^2+^], we found that low [Ca^2+^] correlates with less effective elimination of defects and decreasing spatial correlation lengths ξ (Fig. 3, C and D, and fig. S5, G and H). The anticorrelation between defect annihilation and spatial correlation lengths ξ was observed to persist at both high and low [Ca^2+^] (fig. S5, L and M). Notably, we observed no difference in average cell speed ν when the [Ca^2+^] was altered (Fig. 3E and fig. S5I).

To visualize the spatiotemporal dynamics of ±1 defects in monolayers exposed to EGF at different [Ca^2+^], we mapped the positions of ±1 defects together with the velocity field. At early time points, we observed a buildup of stable ±1 defects at both low and high [Ca^2+^] (Fig. 3F and movies S10 and S11). At high [Ca^2+^], we observed defects that were motile and exhibited effective elimination by defect annihilation leading to instantaneous order (Fig. 3F, top, and movie S10). On the other hand, for cells stimulated under low [Ca^2+^], the defects appeared to be more static, and ± 1 defect pairs were observed to resist elimination by annihilation for longer periods (Fig. 3F, bottom, and movie S11). By tracking ±1 defects over time and calculating their average velocity, we confirmed that defects formed in monolayers stimulated with high [Ca^2+^] move with higher average velocity compared to defects formed under low [Ca^2+^] (Fig. 3G and fig. S6).

**Fig. 4.**
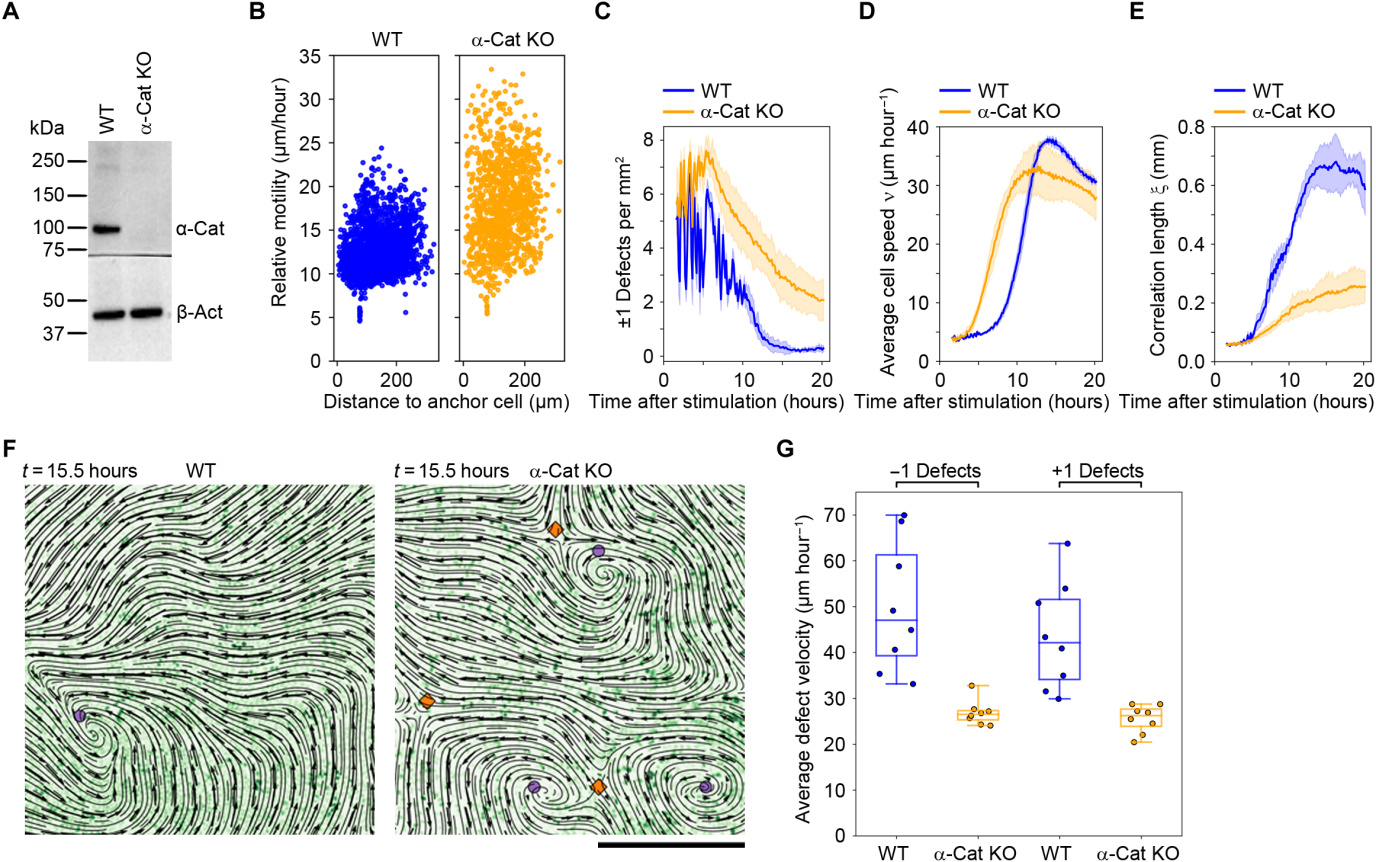
Depletion of α-catenin impedes vortex-antivortex annihilation and polar self-ordering. (**A**) Western blot analysis demonstrating the absence of α-catenin protein expression in CRISPR-edited HaCaT cells. Loading control used was β-actin. (**B** to **G**) WT and α-catenin–depleted HaCaT cells were subjected to serum deprivation for 72 hours before stimulation with FBS. (B) Scatter plots illustrating the relative motility (micrometers per hour) of cells using selected anchor cells as reference points. (C) to (E) Time evolution of ±1 defects (C), the average cell migration speed ν (D), and the spatial correlation length ξ (E) following FBS stimulation at time *t* = 0. The average of eight independent monolayers ± SD is presented. (F) Snapshots of WT and α-catenin–depleted HaCaT cells 15.5 hours after FBS stimulation. Streamline plots generated by PIV and mCherry-tagged histone H2B (green) are depicted. Circles and diamonds indicate computer-based detection of +1 and −1 defects, respectively. Scale bar, 500 μm. See also movies S12 and S13. (G) Boxplot illustrating the average velocity for +1 and −1 defects in FBS-stimulated cells. Average values are shown for *n* = 8 independent monolayers per sample. The whiskers encompass minimum and maximum values. WT, wild type; KO, knockout.

To verify that the observed impact of calcium on defect annihilation and long-range collective cell migration stemmed from its influence on cell-to-cell interactions rather than other calcium-dependent cellular processes, we used CRISPR-Cas9 technology to specifically deplete α-catenin in HaCaT cells (Fig. 4A). This protein is known to play a crucial role in cadherin-mediated cell-to-cell interaction, and depletion of α-catenin has been shown to disrupt cell-to-cell connectivity within epithelial monolayers (*33*). Consistent with this, the depletion of α-catenin resulted in an increase in relative motility in experiments where cells had been subjected to serum depletion and subsequent serum-mediated activation (Fig. 4B). Moreover, α-catenin–depleted cells exhibited a slower elimination of ±1 defects as well as a markedly slower polar ordering of the velocity field (Fig. 4, C to E). Upon closer examination, we observed that the ±1 defects in depleted cells transitioned into a more rigid configuration, characterized by reduced motility and diminished susceptibility to elimination through pair annihilation, compared to control cells (Fig. 4, F and G, and movies S12 and S13). In conclusion, these results suggest that cells enter a phase of high ±1 defect densities at early stages after FBS or EGF-mediated cell sheet activation and that self-ordering through defect annihilation is more effective under conditions where cell-to-cell interactions are stable.

### The emergent velocity field is governed by positional rules for vortices and antivortices

The principle behind topology-guided global polar ordering of an emergent velocity field is based on the ability of the velocity field to first populate itself with local dynamic vortices and antivortices, which can subsequently spread order through the annihilation of defect pairs with opposite charges. For such a global ordering sequence to occur, the defects must comply with a precise set of positional rules during the initial stages of ordering, which depend on the spin orientation of the +1 defect and the relative positioning of oppositely charged defects. For instance, it is crucial that while two +1 defects with opposite spin orientations can be in direct contact, two +1 defects with the same spin orientation must be separated by a −1 defect to prevent the polar motility pattern from becoming frustrated.

**Fig. 5.**
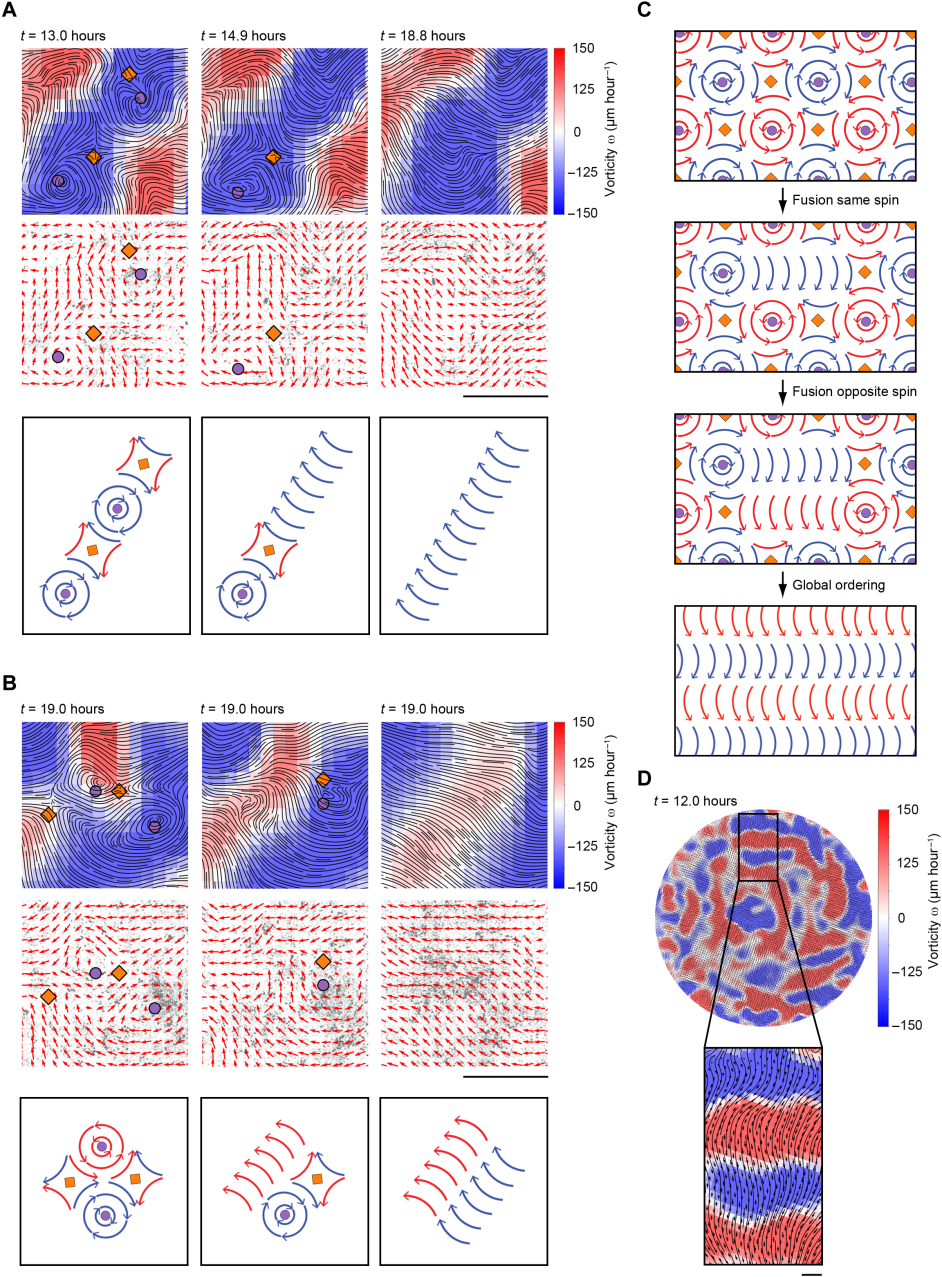
The emergent velocity field is governed by positional rules for vortices and antivortices. (**A**) Annihilation of defect pairs where the +1 defects have the same spin orientation. Top panels show streamline plots in combination with color coded vorticity. Middle panels show the velocity field (red arrows) superimposed on microscopy images of mCherry-tagged cell nuclei. Circles and diamonds indicate computer-based detection of +1 and −1 defects, respectively. Scale bar, 500 μm. Bottom panels show schematic representations of the annihilation events. See also movie S15. (**B**) Annihilation of defect pairs where defect pairs are arranged next to each other and where the +1 defects have opposite spin orientation. Panels are the same as in (A). See also movie S16. (**C**) A model demonstrating the spread of polar order through formation of vorticity arrays and subsequent annihilation of ±1 defect pairs. Circles and diamonds indicate +1 and −1 defects, respectively. (**D**) Wavy patterns formed in HaCaT keratinocyte monolayers at full polar order 12 hours after serum stimulation. A zoomed-in region showing characteristic waves is highlighted. The images show streamline plots of the velocity filed in combination with the vorticity field, represented by red and blue colors. Scale bar, 500 μm.

To identify such positional ordering rules in our cell-based system, we examined the spin orientation of +1 defects by mapping the vorticity ( $\vec{\omega }=\vec{\nabla }\times \vec{u}$ ), in which counterclockwise and clockwise vorticity are represented by blue and red color, respectively. By observing the behavior of a substantial set of ±1 defect pairs (*n* ≥ 50, movie S14), we established that the annihilation between oppositely charged defects is only observed within arrays of ±1 defect pairs, where all the +1 defects have the same spin orientation (Fig. 5A and movie S15). When two neighboring +1 defects with opposite spin orientation annihilate with their respective −1 defects, they merge their regions with an ordered velocity direction polarity (Fig. 5B and movie S16). This leads to the propagation of polar order along arrays of defect pairs with the same +1 spin orientation and the fusion of polar order between rows of alternating clockwise and counterclockwise spin orientations in the perpendicular direction, as depicted in Fig. 5C.

The local vorticity of the velocity fields was observed to persist even after annihilation-mediated ordering had occurred (Fig. 5, A to C). In agreement with this, we readily observed wavy patterns in the velocity field following serum-activated ordering of HaCaT keratinocytes, representing memories of previous arrays of vortices (Fig. 5D).

Collectively, our observations affirm an ordering regime in which the initiation of migration within an otherwise static and disordered epithelial monolayer results in the colonization of the nascent velocity field by ±1 defect pairs. These defect pairs subsequently engage in paired annihilation events, orchestrated by precise positioning and annihilation rules dictated by the spin orientation of the +1 defects. This orchestration culminates in the establishment of structured, polarized velocity fields characterized by undulating patterns that preserve recollections of preceding vortices.

### Reversal of motility direction is facilitated by topology-guided polarity flipping

**Fig. 6.**
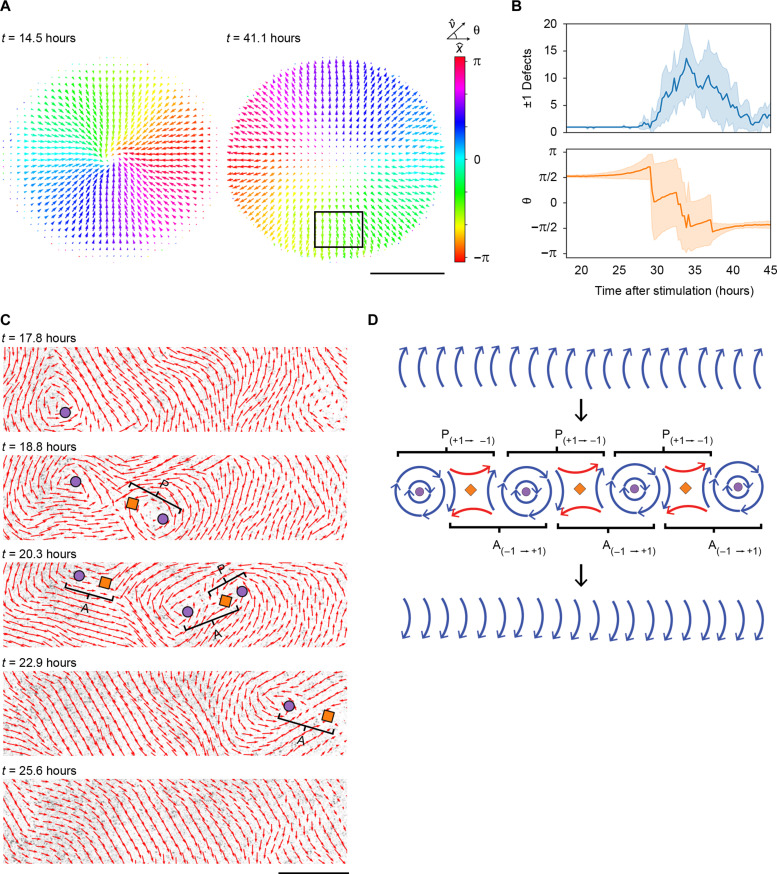
Reversal of motility direction is facilitated by topology-guided polarity flipping. (**A**) Snapshots of vector fields derived from PIV at 14.5 and 41.1 hours after serum stimulation, representing the average from eight separate monolayers. The color map represents the angle of the local velocity field relative to the *x* axis. Scale bar, 2 mm. (**B**) The number of ±1 defects detected in the entire monolayer (top) and the angle θ (in π) of the migration orientation relative to the *x* axis in a selected region (indicated by the rectangle in A) of the monolayer (bottom). (**C**) Time series showing proliferation, defect partner exchange, and annihilation events that lead to reversed cell motility direction. The images show the velocity field (red arrows) superimposed on fluorescence microscopy images of cell nuclei (mCherry-tagged histone H2B). Circles and diamonds indicate computer-based detection of +1 and −1 defects, respectively. Scale bar, 500 μm. See also movie S17. (**D**) A schematic representation of topology-mediated polarity flipping. Strings of interconnected +1 and −1 defects, where all +1 defects have the same spin orientation, are formed along regions of uniform vorticity. The polar orientation of the velocity field is reversed by switching the defect pairing configuration. (C and D) P and A stands for proliferation and annihilation, respectively.

In serum-stimulated HaCaT monolayers, confined to the round bottom surface of 96-well plates, cells migrate toward the well center, forming a radial density gradient. To counteract the buildup of high cell densities at the center of the well, cell migration direction is reversed at late time points, leading to migration in the opposite direction away from the well center (Fig. 6A). By comparing the time evolution of motility reorientation at a local region (marked by a rectangle in Fig. 6A) and the occurrence of ±1 defects, we found that reversal of collective migration polarity coincides with a transient increase in these defects (Fig. 6B). Furthermore, by mapping defects and vorticity during reorientation of cell migration, we discovered interconnected strings of +1 and −1 defects that emerged from regions of uniform vorticity orientation at the time of collective motility reversal (fig. S7). Moreover, by performing time-lapse analysis of the transiently appearing vortex-antivortex pairs, we observed that individual defects are both repelled by their original pairing partners and attracted to a partner of opposite charge from a neighboring defect pair. As a result, when defect pairs annihilate after the partner switch, the polar order generated consistently leads to collective migration in the opposite direction (Fig. 6C and movie S17). These observations are consistent with a theoretical model in which strings of ±1 defects form along elongated regions of uniform vorticity orientation. The newly formed defects subsequently have the potential to switch from a +1 → −1 pairing configuration to a −1 → +1 pairing configuration, resulting in polar order of the velocity field in the opposite direction (Fig. 6D). Thus, the transient proliferation and annihilation of ±1 defect pairs within conserved vorticity domains represent an effective strategy for collectively reorienting cell migration.

## DISCUSSION

The present study addresses a fundamental question related to the behavior of biological tissues: How do immobile cells within a tissue, lacking any specific alignment or direction, transit into a dynamic cell collective characterized by a uniform migration direction? The mechanism described in this study suggests that long-range order in an emergent field of migrating cells is not achieved through long-distance cell communication. Instead, cells have an intrinsic ability to create and adapt to local topological defects, which follow predetermined arrangements based on the constraints and spin orientation of these defects. Long-range order then follows naturally through the ability of defects of opposite charges to become attracted to each other and annihilate. The experimental findings and numerical simulations presented in this study bring to light several rules, such as the consistent reversal of cell migration direction between neighboring defects of opposite charge following annihilation, and the mandatory presence of a −1 defect between two +1 defects with the same spin orientation. In addition to the potential of generating long-range order, these ordering rules also explain the wavy sinusoidal pattern observed in the velocity field after full order is achieved and how ±1 defects contribute to a 180° reversal of cell motility direction. It is remarkable that our experiments using living cells in a circular confinement within an AES yield nearly identical results as an experimental setup involving the same geometric constraints and activation of particle self-propulsion in a hydrodynamic system with colloidal particles (*21*). This observation emphasizes the universal nature of topology-guided self-ordering across a diverse range of active matter systems. An important aspect to consider is that both the previously published studies on colloid particles and our cell-based system demonstrate motility-induced ordering mechanisms. This feature may hold critical significance in comprehending the emergence of ±1 defects, followed by their subsequent untangling into a uniform polar orientation.

To achieve full order through ±1 defect annihilation, it is reasonable to assume that several conditions must be met. In the present study, we found that the ability to self-organize into a long-range polar order is affected by the ability of cells to become organized into a solid cell sheet and that the systematic reduction in cell-to-cell affinity leads to progressively slower self-ordering. In agreement with this, our experimental observations are effectively replicated by an AES model. It is worth noticing that both our experimental observations and our numerical simulations demonstrate that ordering of the system is initiated at low initial velocity. Unexpectedly, this initial motion is what drives the polarized organization of cell migration. On the basis of this observation, it may be more accurate to describe the process as force synchronization rather than motion synchronization. That is, the cells sense the forces exerted by neighboring cells and adjust their direction of propulsion accordingly, leading to the emergence of global motion. This appears to be an effective way to unjam and induce global motion without requiring substantial initial movement.

In addition to the ability of keratinocytes to form an elastic solid material, several other parameters may affect the process of polar ordering in epithelial monolayers. For instance, keratinocytes may have an intrinsic ability to steer into vortices, as suggested by studies showing that single keratinocytes or pairs of keratinocytes tend to migrate in a rotating pattern (*34*). The capacity of cells to self-organize into a field of collective cell migration may also be influenced by the ability of cells to regulate intercellular tension. Notably, a previous study from our lab has demonstrated that stress amplification in the form of supracellular tension and traction forces immediately after serum stimulation is critical for activation of coherent motion, when cells are plated on a soft acrylamide substrate (*23*). In this respect, it is worth mentioning that tension has been shown to influence the dynamic behavior of an active solid system (*35*). We are now investigating how mechanical forces such as traction forces, intercellular tension, as well as extensile and compressive forces affect the dynamic behavior of ±1 defect pairs after serum stimulation of quiescent HaCaT keratinocytes. Last, although this study primarily focuses on polar activities, it should be emphasized that epithelial tissues also exhibit nematic order and nematic defects, such as the +1/2 and −1/2 defects that have been shown to contribute to turbulence in systems with living cells (*36*, *37*). Although our numerical simulations using the AES model, as well as studies on activated colloid particles, show that the polar ordering of an emergent velocity field through the guidance of integer topological defects can occur independent of a nematic component, further investigations into the interconnection between polar and nematic matter will be important for a comprehensive understanding of tissue organization and dynamics.

The process of polar ordering achieved through the annihilation of vortex-antivortex pairs is well-established in nonliving materials like magnetic fields and colloid particles. Our study reveals that this generic mechanism of topology-guided self-organization also plays a vital role in generating long-range polar order within biological tissues, such as epithelial monolayers. Consequently, these findings explain how a static quiescent epithelial monolayer with initially disordered polarity effectively emerges toward a dynamic state with long-range ordered polarity. Our discoveries carry significant implications, not only for understanding the emergence of long-range coherent dynamics of biological tissues but also for comprehending large-scale self-organization of AESs in general.

## MATERIALS AND METHODS

### Activation of quiescence-dependent self-ordering

The experimental approach used to induce quiescence-dependent activation of collective migration has been previously described (*23*, *24*). Immortalized human keratinocytes (HaCaT) (*38*) or HaCaT cells expressing mCherry-tagged histone H2B (*24*) were cultured in Iscove’s modified Dulbecco’s medium (MedProbe) supplemented with 10% fetal bovine serum (FBS; Thermo Fisher Scientific) and penicillin/streptomycin (90 U/ml; Lonza). For each experiment, 96-well plates (Greiner Sensoplate; M4187-16EA, Merck) or 12-well plates (P12G-1.5-14-F; MatTek Corporation) were coated with collagen IV (20 μg/ml; C7521, Merck) before seeding the cells at 75,000 or 600,000 cells per well, respectively. The cells were then cultured in normal growth medium at 37°C and 5% CO_2_ overnight. To induce quiescence, the cells were serum deprived for 72 hours before being activated with normal growth medium containing 15% FBS. For experiments involving the manipulation of calcium concentration, the serum-deprived cells were stimulated with CnT-Prime Epithelial Proliferation Medium (CELLnTEC), a low calcium medium, human recombinant EGF (10 ng/ml; 236-EG, R&D Systems), and the indicated concentrations of calcium.

### Live-cell imaging

Image acquisition of cells plated in 96-well plates was performed using the ImageXpress Micro Confocal High-Content microscope controlled by the MetaXpress 6 software (Molecular Devices). Wide-field images were acquired using a Nikon 4×/0.2 numerical aperture (NA) PLAPO air objective at pixel binning 2, with a filter set for mCherry fluorescence detection, and an environmental control gasket that maintains 37°C and 5% CO_2_. Four tiled images were acquired per well every 4 or 16 min for 30 to 50 hours. The acquired time series were then processed by stitching (using the MetaXpress software) to generate movies representing the entire well bottom surface.

For cells plated in 12-well plates, we used a Zeiss Axio Observer.Z1 wide-field microscope. Time-lapse images were acquired using a Zeiss 5×/0.16 NA EC Plan-Neofluar air objective with phase contrast, transmitted light, and a CO_2_ incubation chamber maintaining 37°C and 5% CO_2_. Four tiled images at the center of the well were acquired every 4 min for a total imaging period of approximately 48 hours. Images from each well were stitched together before analysis.

Imaging of scratch assays was performed using a Nikon CrestOptics spinning disc confocal microscope equipped with a Nikon Plan Fluor DL 10× Ph1 air objective, RFP fluorescence channel, and a CO_2_ incubation chamber maintaining 37°C and 5% CO2. HaCaT cells expressing mCherry-tagged histone H2B were seeded in 12-well plates. Cells were starved in serum-depleted medium for 72 hours, and a horizontal scratch was introduced into the monolayer immediately before serum stimulation. A 4 × 3 grid of images was acquired every 8 min for a total imaging period of approximately 50 hours. Images from each well were stitched together before analysis using the NIS-Elements software. For all microscopy time-lapse experiments, image acquisition started 1 hour after serum stimulation.

### Calculation of cell velocity

To calculate the velocity field using particle image velocimetry (PIV), we used a Python script that used the OpenPIV package. Our raw data input consisted of time series of either mCherry-tagged histone H2B (nuclei label) or phase contrast images (visualize cell contour). The interrogation window size was set to 165 μm with a 99-μm overlap between consecutive windows. The average cell speed ν was extracted from the PIV data using the script Defects.py available in the Zenodo repository.

Particle tracking was carried out using the TrackMate plugin in ImageJ. Particle tracking was performed on mCherry-tagged nuclei over a time period of 30 hours, comprising 450 frames. The average cell speed per frame was calculated using the python script TrackMate_speed_data.py available in the Zenodo repository.

### Detection and analysis of +1 and −1 defects

The ±1 defects characterized by their local winding number were identified on the basis of the velocity field obtained using PIV. A Python script was used to calculate the local winding number for each position in the velocity field, based on the following equations

$$\omega_{i,j}=\frac{1}{2\pi }\sum_{k=1}^{8}\delta_{k}$$

δk=Δk+2π,Δk<−πΔk,−π<Δk<+πΔk−2π,Δk>+π

Δk=α1−α8,k=1αk−αk−1,k>1

αk=θk+2π,θk<0θk,θk≥0

$$\theta_{k}=\text{atan}2\left(y_{k},x_{k}\right)$$

Here, ω_*i*,*j*_ is the winding number, δ*_k_* is the range-adjusted velocity direction difference, Δ*_k_* is the velocity direction difference, α*_k_* is the range-adjusted direction, and θ*_k_* is the direction of the velocity with components (*x_k_*, *y_k_*). These values are calculated for the cells in each of the eight pixels *k* ∈ (1,8), surrounding the central pixel (*i*, *j*) in an anticlockwise manner, starting at the top left cell. These calculations are based on an equation by Chardac *et al.* (*22*), and the velocity directions θ*_k_* are indicated in their fig. S4B.

We only considered defects with a charge of +1 or −1 that persisted for at least two consecutive time frames. To avoid counting the same defect multiple times, we merged defects that were closer than 165 μm into a single defect. The defects and their mapped positions were retrieved using the script Defects.py, which is available in the Zenodo repository.

### Calculation of defect velocity

To determine the velocity of defects in stimulated monolayers, we used the TrackMate plugin in ImageJ to track the coordinates of +1 and −1 defects. Our analysis included only stable tracks spanning at least 25 consecutive time points. To calculate defect velocity, we computed the Euclidean distance traveled by each defect and divided it by the number of time points in the corresponding track. The average defect velocity was then obtained as the mean of all defect velocities measured from a single well.

### Calculation of spatial correlation length

The spatial correlation function *C*(*r*) measures how similar the velocity vectors are at different locations and distances within a 2D velocity field. The function is defined as the average inner product of the velocity fluctuations at a distance *r* apart, weighted by the cosine of the angle between the velocity vectors at each location

$$\begin{array}{c} C\left(r\right)=\frac{1}{\left[N_{x}*N_{y}*\left(N_{x}-r\right)\right]}\\ \sum \sum \left[\delta u_{\left(x,y\right)}\delta u_{\left(x+r,y\right)}+\delta v_{\left(x,y\right)}\delta v_{\left(x+r,y\right)}\right]\;\text{cos}\left[\theta_{\left(x,y\right)}-\theta_{\left(x+r,y\right)}\right] \end{array}$$

where *N_x_* and *N_y_* are the dimensions of the velocity field, δ*u*_(*x*,*y*)_ and δ*v*_(*x*,*y*)_ are the *x* and *y* components of the velocity fluctuations at position (*x*, *y*), *r* is the distance between two positions (*x*, *y*) and (*x* + *r*, *y*), and θ_(*x*,*y*)_ is the angle of the velocity vector at position (*x*, *y*). The double summation ΣΣ is taken over all positions (*x*, *y*) in the velocity field such that *x* + *r* < *N_x_*.

To find the correlation length ξ, the correlation function *C*(*r*) was normalized, and an exponential curve was fitted to it. The point at which the fitted curve crosses a threshold value of 0.5 (which is the halfway point between the maximum and minimum values of the normalized correlation function) was taken as the correlation length. An interpolation function was used to estimate the distance at which the normalized correlation function equals 0.5. The correlation length ξ was retrieved using the script Spatial_correlation.py available in the Zenodo repository.

### Calculation of vorticity

Vorticity ω was calculated using the following equation

$$\omega =\frac{\delta v}{\delta x}-\frac{\delta u}{\delta y}$$

where ω is the vorticity and $\frac{\delta v}{\delta x}$ and $\frac{\delta u}{\delta y}$ are the partial derivatives of *v* with respect to *x* and *u* with respect to *y*, respectively. The vorticity ω was retrieved using the script Vorticity.py available in the Zenodo repository.

### Calculation of cell shape orientation

For the determination of tensor maps illustrating the mean local orientation of cell shapes within the velocity field, we acquired microscopy images of live cells using a Zeiss Axio Observer.Z1 microscope equipped with a 10× phase contrast objective. Computation of tensor maps was accomplished through the utilization of the Orientation J plugin in ImageJ, following a previously published methodology (*13*). The computer script Tensor_velocity_angles2_batch.py, which was used for global quantification of velocity direction versus cell shape tensor orientation, is available in the Zenodo repository.

### Calculation of relative cell motility

Visualization and analysis of relative dynamics of neighboring cells in keratinocyte monolayers, with various cell-to-cell affinities, were carried out using ImageJ macros and Python-based scripts. In brief, the TrackMate plugin in ImageJ was used to generate regions of interest in randomly selected sites in the mCherry channel (displaying histone H2B–tagged cell nuclei) of time-lapse image series (*39*). Subsequently, an ImageJ macro that performs image-based registration generated 250 × 250 pixel images keeping a specified cell nucleus in the center of each image. The time-lapse series generated were 101 frames in length representing a time period of 6.7 hours of live-cell imaging. After image-based registration, the trajectories of cells, in relation to the anchor cell at the center of each image, was analyzed by particle tracking using TrackMate. The relative motility (micrometers per hour) for each cell was calculated as the sum of distance traveled per frame divided by the total time in hours. Relative motility = ∑*d_n_*/hour, where *d* is the distance traveled for *n* frames.

### Analysis of cell diffusion

Time-lapse videos of mCherry-tagged histone H2B expressing cells were analyzed by particle tracking, using the TrackMate plugin in ImageJ. Subsequently, Python-based scripts were used to identify pairs of Track-IDs, where cells were within a 30-pixel distance (about 23 μm) in the initial frame. For each Track-ID pair, the squared distance was calculated per frame, and the overall average squared distance for all Track-ID pairs was determined for a time period of 16 to 21 hours after stimulation. This is the time period when cells displayed the highest average cell speed. Linear regression was performed to fit a linear model to the curve for each sample and determine the slope of the tangents. The scikit-learn Python package was used for this purpose. At least 30 Track-ID pairs were analyzed per sample.

### Genome editing using CRISPR-Cas9

HaCaT cells expressing mCherry-tagged histone H2B were engineered to knockout the *CTNNA1* gene using Cas9 (Synthego) and a pool of synthetic guide RNA (sgRNA; Synthego) composed of the following sequences: sgRNA#1, uuguaggaauugaaagaugu; sgRNA#2, gauccucuauacugcauccc; and sgRNA#3, auacaagcagcugcagcagg. Cas9 and sgRNA were mixed at a 1:9 ratio before mixing with cells resuspended in 3P primary cell nucleofactor solution [5 mM KCl, 15 mM MgCl_2_, 90 mM NaCl, 10 mM glucose, 0.4 mM Ca(NO_3_)_2_, 0.4 mM CaCl_2_, 40 mM Na_2_HPO_4_/NaH_2_PO_4_ (pH 7.2)]. Electroporation was subsequently performed using a Lonza 4D Nucleofactor, program CM-137 (Lonza). Following 1 week of cell culturing, validation of gene depletion was performed using three methods: (i) Polymerase chain reaction–mediated genotyping; two sets of primers were used: primer set#1, ggtctgtgtgattccaaagctcaga and gccttgatcacttttccccagaaatctg; and primer set#2, cttctttgacagaaggggaaccttagc and aagaatgacctcataggaaacacaagctac; (ii) DNA sequencing followed by sequence analysis using the Synthego CRISPR-Performance analysis software; and (iii) Western blotting using an α-catenin–specific antibody (alpha-CAT-7A4; 13-9700, Thermo Fisher Scientific) and β-actin antibody (AC-15; sc-69879, Santa Cruz Biotechnology) for loading control.

### Numerical simulations

When performing numerical simulation using the AES model described by Eqs. 1 and 2, the spring force between cells is modeled by an elastic energy with a harmonic term giving a linear force contribution and a fourth-order term that gives a nonlinear correction for large deformations

$$U\left(r_{\mathrm{ij}}\right)=\frac{K}{2}{\left(d_{\mathrm{ij}}-r_{\mathrm{ij}}\right)}^{2}+B{\left(d_{\mathrm{ij}}-r_{\mathrm{ij}}\right)}^{4}$$ (3)

where *r_ij_* is the distance between two neighboring beads and *d_ij_* is the equilibrium distance. The nonlinear term suppresses compressional instabilities in the solid and avoids overstretching of links between neighboring beads. The parameters in the model are cell propulsion velocity *V_c_* (μm hour^−1^), cell size *d* (μm), elastic spring constant $K\left(\frac{N}{\mu m}\right)$ , cell turning rate $\gamma \left(\frac{1}{\mu m}\right)$ , and the cell-substrate friction coefficient $\zeta \left(\frac{N\;h}{\mu m}\right)$ . The cell size and propulsion velocity is derived from experiments, and, in the model, we set the average distance between cells to *d* ≈ 22.5 μm and *V_c_* = 35 μm hour^−1^. There are two effective parameters in the model, the dimensionless spring constant: $\tilde{K}=\frac{\mathrm{Kd}}{\zeta V_{c}}$ and dimensionless turning rate: $\tilde{\gamma }=\gamma$ . The dimensionless spring constant determines the rate of compression in the system. If $\tilde{K}\ll 1$ , then strain becomes large, leading to very large deformations in the monolayer. We find that simulations with values in the range $\tilde{K}=10\;\text{to}\;50$ give moderate deformations. Likewise, for the turning rate, we find that values $\tilde{\gamma }≳1$ will give polar ordering of the system. The equilibrium distance is uniformly distributed on the interval *d_ij_*∈ [0.8*d*, 1.2*d*], which ensures microscopic lattice disorder and global isotropic elasticity. At the boundary, we have a repulsive spring force to keep cells in confinement, and the cell polarity turns away from the boundary. The radius of the circular confinement is set to *R* = 3.5 mm, and the number of cells is *N* = 100,000. To prepare the bead-spring networks, isotropic assemblies of points (“cells”) were created by Monte Carlo simulation of growing differently sized and repulsive disks, and the connection network (springs) between points was created by Voronoi tessellation (*30*).

## Supplementary Material

20240417-1

## Notes

**Correction (1 May 2024):** Due to a production error, the *y* axis label in Fig. 1I was incorrect. “±1 Defect annihilation” has been corrected to “±1 Defect proliferation.” The online and PDF versions of the article have been corrected to include the updated figure.
