# Supplementary material for: Topology-guided polar ordering of collective cell migration
Source: Sci Adv. 2024 Apr 17;10(16):eadk4825. doi: 10.1126/sciadv.adk4825 (PMC11023523; doi:10.1126/sciadv.adk4825)
Supplement: Supplementary file 2 — Figs. S1 to S7 Table S1 Legends for movies S1 to S17 [file sciadv.adk4825_sm.pdf]

Science Advances
AAAS

Supplementary Materials for

Topology-guided polar ordering of collective cell migration

Emma Lång *et al.*

Corresponding author: Stig Ove Bøe, s.o.boe@medisin.uio.no

Sci. Adv. **10**, eadk4825 (2024)
DOI: 10.1126/sciadv.adk4825

The PDF file includes:

Figs. S1 to S7
Table S1
Legends for movies S1 to S17

Other Supplementary Material for this manuscript includes the following:

Movies S1 to S17

Figures

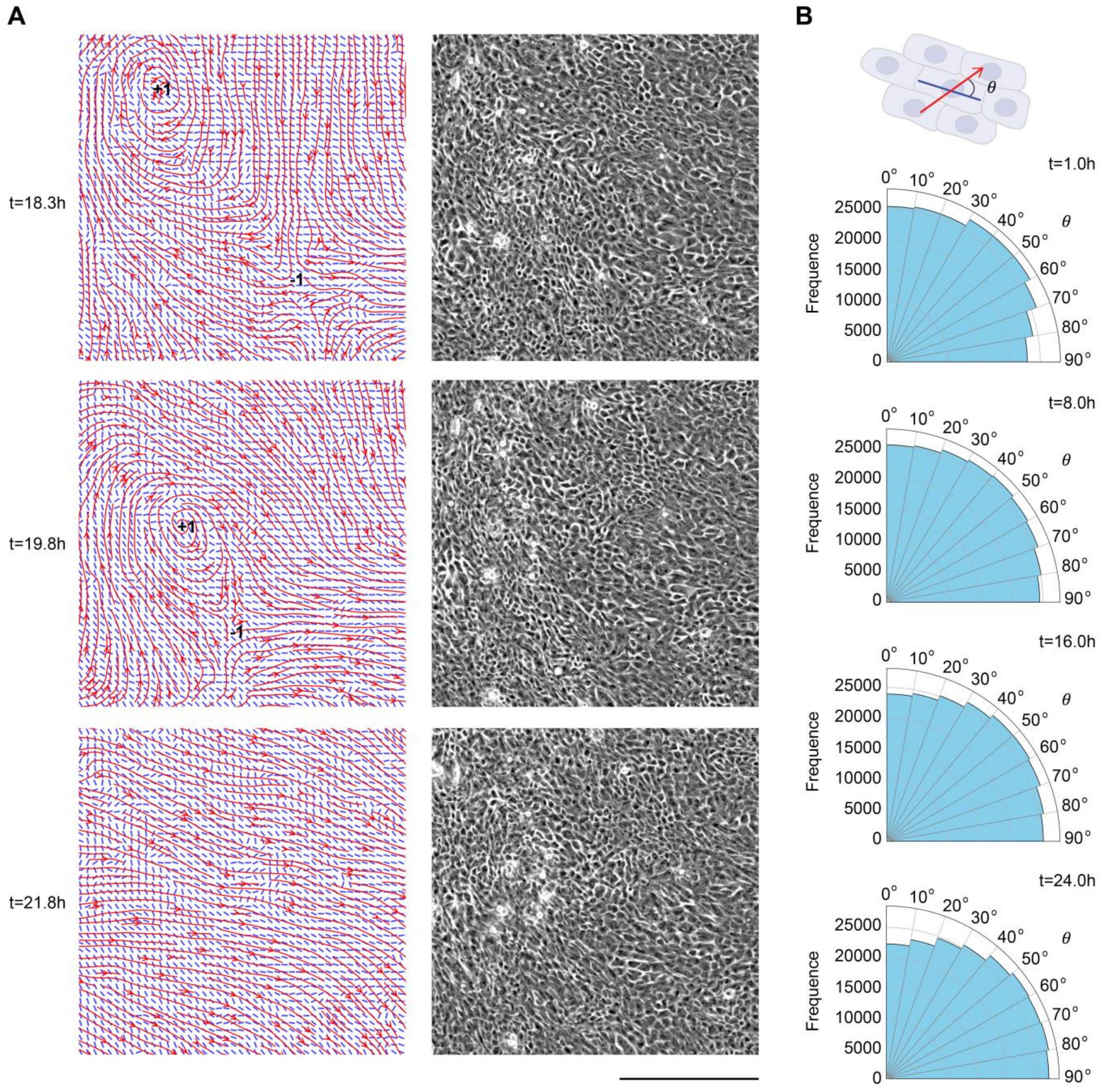

Fig. S1. Comparison of cell migration direction and cell shape orientation

(**A**) Left panels show the velocity field (represented by red stream lines generated by PIV) and average local cell orientation (represented by blue tensor orientation maps generated using OrientationJ). Right panels show the corresponding phase contrast images. The three different time points feature a ±1 defect pair that generates instantaneous order in the velocity field through defect annihilation. Scale bar, 200 µm. (**B**) Quantification of cell shape orientation versus migration direction overtime. The schematic in the top panel shows how the angle (θ), representing the angle between velocity direction (red arrow) and shape orientation (blue line), is determined. Radial histograms show the frequency of specific values of θ at four selected time points after serum stimulation of quiescent cell sheets. The histograms represent the sum of angles collected from n = 15 separate microscopy fields of view (field of view size = 1608 x 1608 µm).

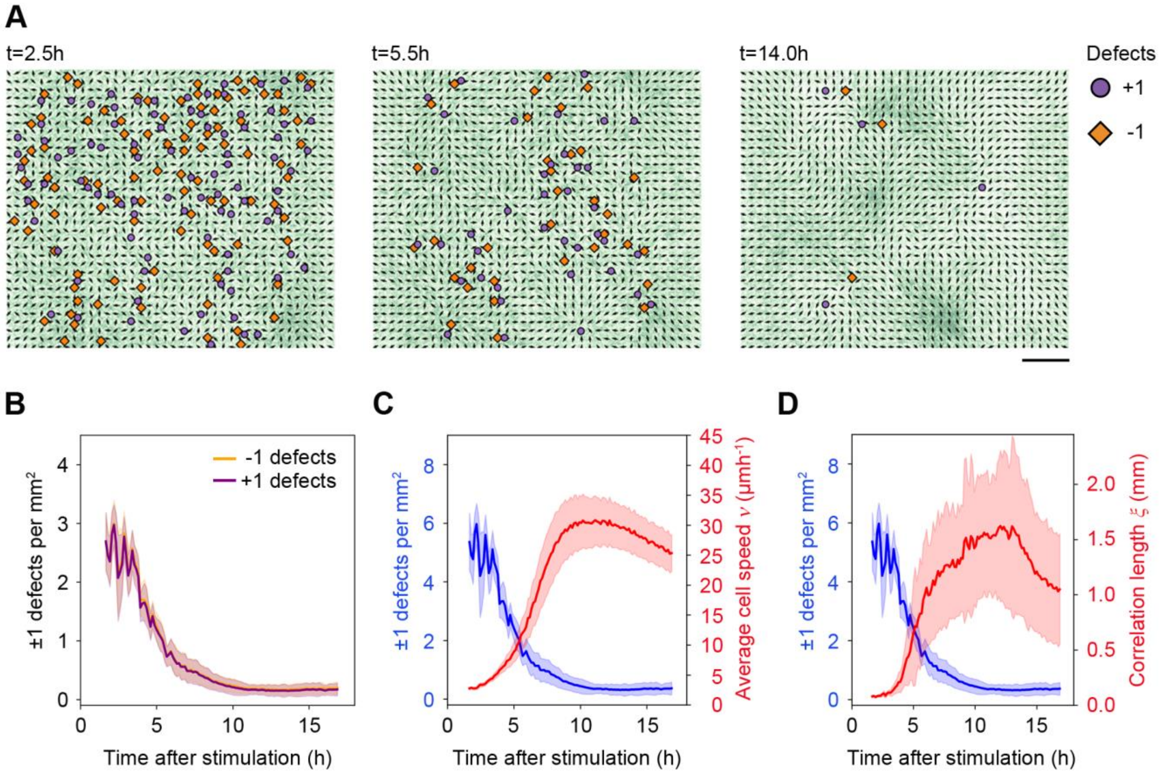

Fig. S2. Motility-induced self-ordering using squared confinement

(**A**-**D**) HaCaT cells were seeded in 96-well plates with squared shaped wells and subjected to serum deprivation for 72 h followed by serum activation at t=0h. (**A**) Detection and mapping of ±1 defects within the emergent velocity field. Nuclei are labeled with mCherry-tagged Histone H2B (green), and the normalized vector field shows the direction of cell migration. Circles and diamonds indicate computer-based detection of +1 and -1 defects, respectively. Scale bar, 1 mm. See also movie S6. (**B**) Time evolution of -1 and +1 defect densities after serum activation. (**C**) Time evolution of ±1 defect densities (blue line) and the average cell migration speed *v* (red line) after serum activation. (**D**) Time evolution of ±1 defect densities (blue line) and the spatial correlation length ξ (red line) after serum activation. (**B**-**D**) Graphs represent average values ± SD from n=8 separate monolayers.

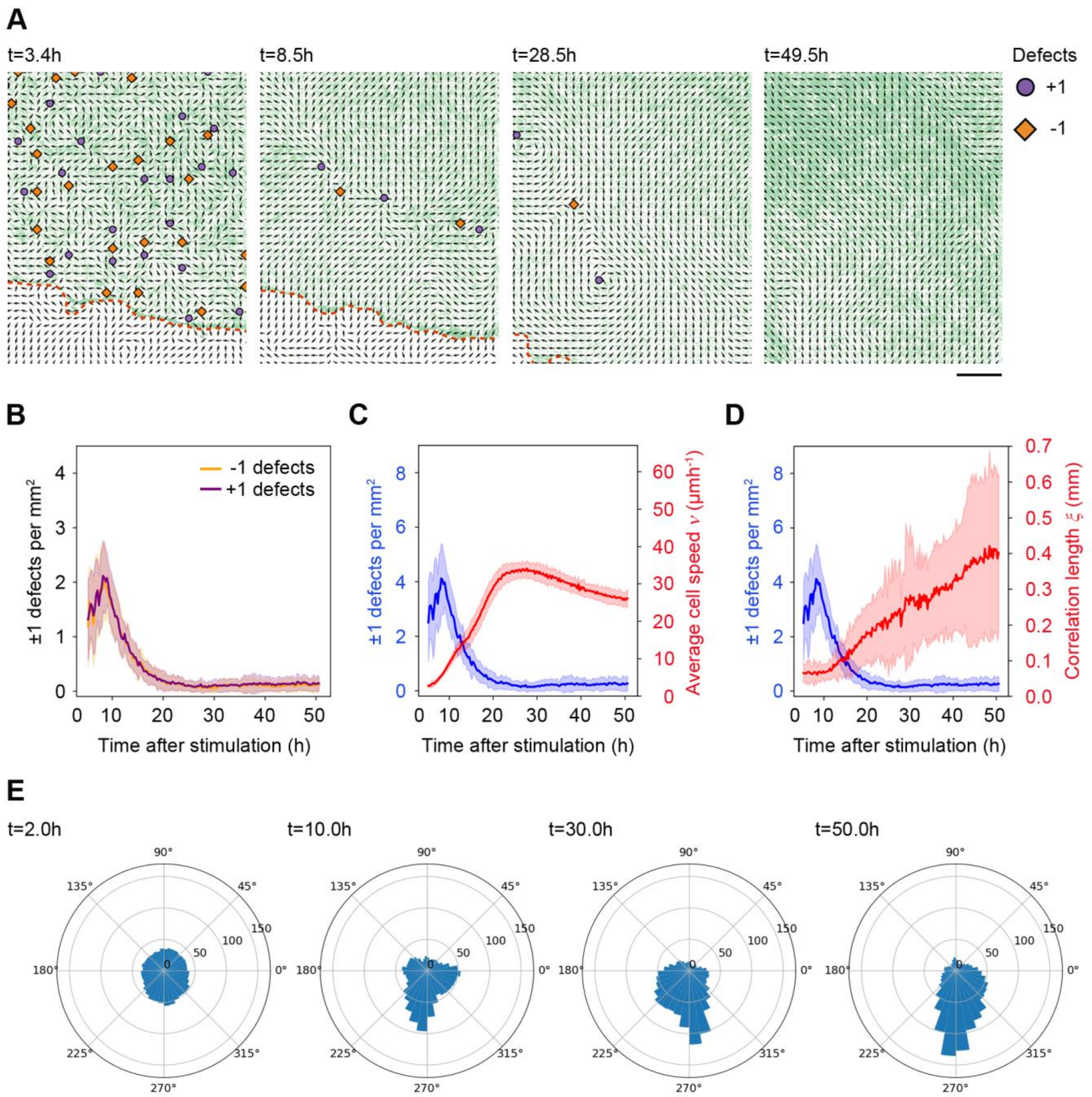

Fig. S3. Motility-induced self-ordering in the vicinity of a wound edge
(**A**-**D**) HaCaT cells were seeded in 12-well plates and subjected to serum deprivation for 72 h followed by serum activation at t=0h. A scratch wound was introduced to each monolayer immediately prior to FBS stimulation. (**A**) Detection and mapping of ±1 defects within the emergent velocity field. Nuclei are labeled with mCherry-tagged Histone H2B (green), and the normalized vector field shows the direction of cell migration. Stapled red lines indicate the wound edge. Circles and diamonds indicate computer-based detection of +1 and -1 defects, respectively. Scale bar, 500 µm. See also movie S7. (**B**) Time evolution of -1 and +1 defect densities after serum activation. (**C**) Time evolution of ±1 defect densities (blue line) and the average cell migration speed v (red line) after serum activation. (**D**) Time evolution of ±1 defect densities (blue line) and the spatial correlation length ξ (red line) after serum activation. (**B**-**D**) Graphs represent average values ± SD from n = 32 individual fields of view, with each field of view encompassing an area of 2636 x 2636 µm. (**E**) Radial histograms displaying the angles of each vector within the velocity field relative to the wound edge,

which is aligned with the x-axis. Each diagram represents the average of n = 32 individual fields of view, with each field of view encompassing an area of 2636 x 2636 µm.

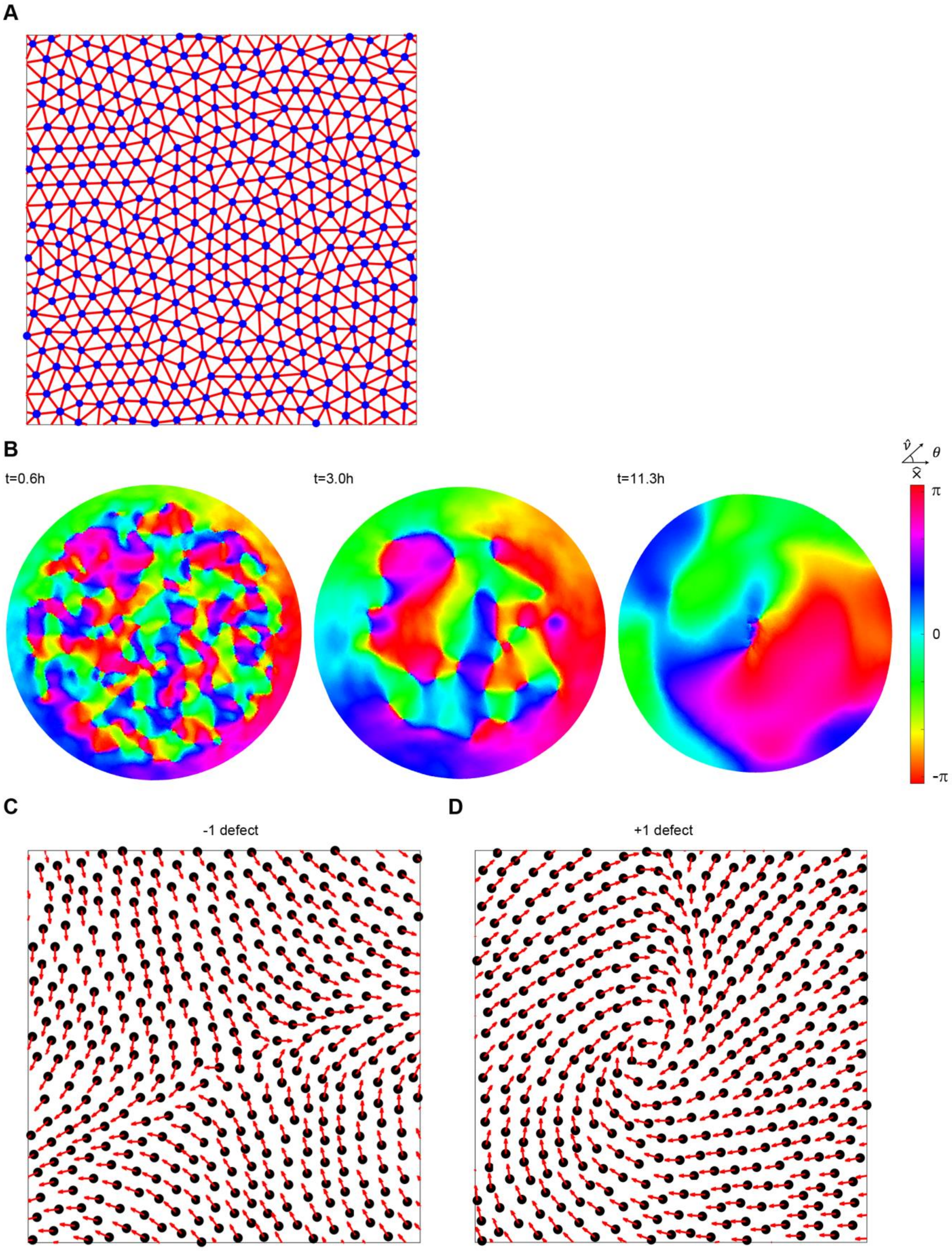

Fig. S4. Numerical simulations using the AES model
(**A**) Zoomed in snapshot of the bead-spring network used in numerical simulations. The network is created by equilibration of repulsive beads (energy minimization) of size in the range (0.8,1.2) d. The spring network is created by subsequent Delaunay triangulation of particle positions. (**B**) Time evolution of polar ordering in numerical simulations. The field contains N=100000 particles. (**C**) Zoomed in snapshot showing a -1 defect. Arrows represent

the normalized velocity field on top of individual particles. (**D**) Zoomed in snapshot showing a +1 defect. Arrows represent the normalized velocity field on top of individual particles.

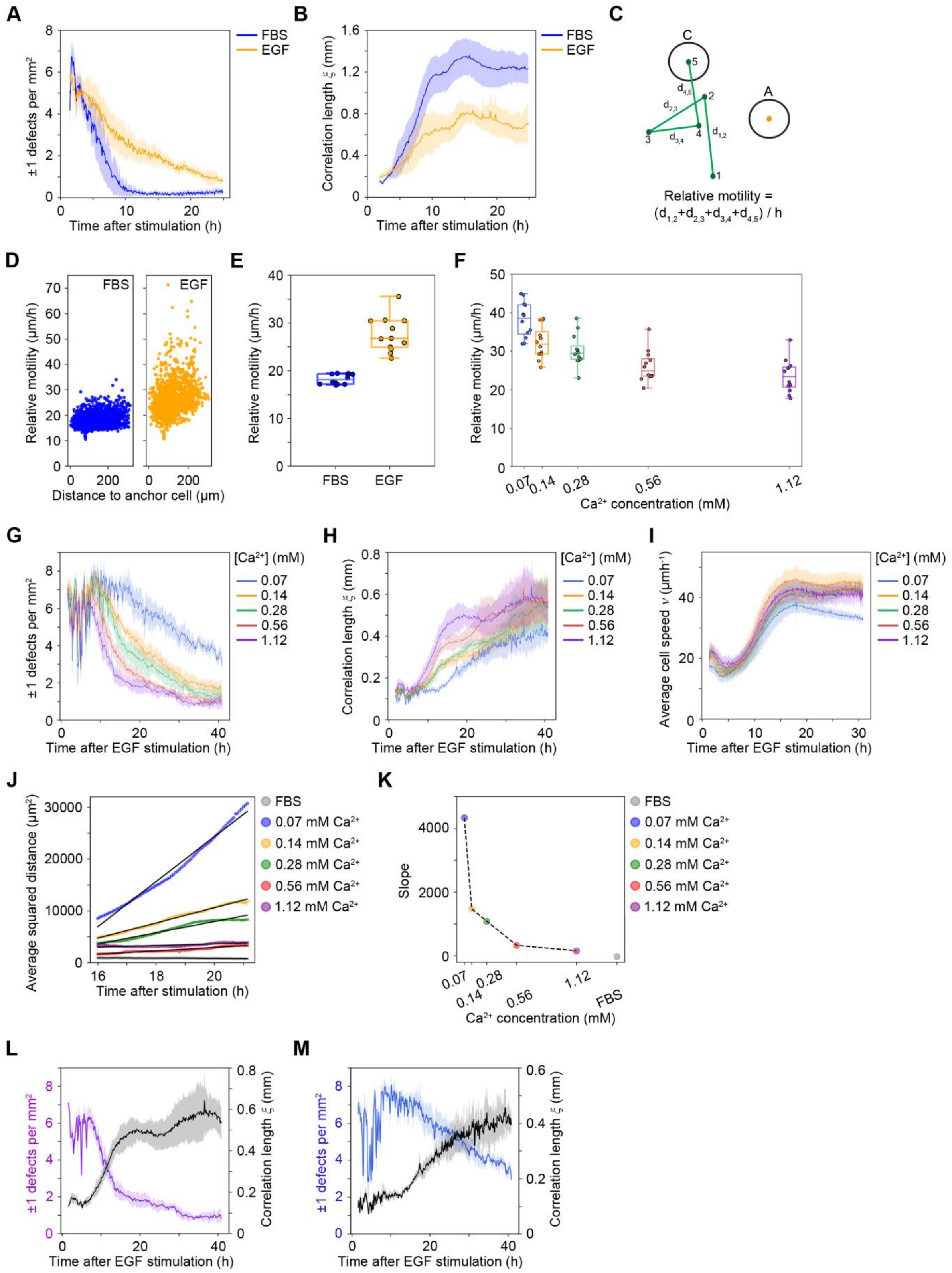

Fig. S5. The effect of intercellular stability on defect annihilation and monolayer self-ordering

(**A**-**B**) Time evolution of ±1 defects (**A**) and the spatial correlation length ξ (**B**) is shown for monolayers exposed to FBS or EGF at time t=0h. For each sample, average values ± SD from n = 8 separate monolayers are shown. (**C**) Schematic describing the calculation of relative motility for cells in relation to the anchor cell in a dataset. The anchor cell, A, is locked in the

center of the field of view by image registration. Neighboring cell, C, trajectories are determined using particle tracking (TrackMate) and the total length traveled per hour calculated (µm/h) as depicted. (**D**) Scatter plots depicting the relative motility (µm/h) of cells in relation to the anchor cell after treatment with FBS or EGF. Analysis was performed on n=12 field of view per sample. (**E**) Boxplot depicting the relative motility (µm/h) of cells in relation to the anchor cell after treatment with FBS or EGF. Each data point in a boxplot represents the average relative motility per field of view. Analysis was performed on n = 12 fields of view per sample (same dataset as in **D**). (**F**) Boxplot depicting the relative motility (µm/h) of cells in relation to the anchor cell after treatment with EGF and different Ca^{2+} concentrations. Analysis was performed on n=12 field of view per sample (same dataset as in Fig. 3B). Whiskers include minimum and maximum values. (**G-I**) Time evolution of ±1 defects (**G**), the spatial correlation length ξ (**H**), and average cell speed v (**I**) is shown for the different Ca^{2+} concentrations tested. Average values ± SD are presented for n=8 separate monolayers. (**J**) Line plot showing the average squared distance (μm^2) for pairs of migrating cells (n>30) plotted for the time period of 16 to 21 h after stimulation, when cells displayed the highest average cell speed. Linear regression was performed to fit a tangent (black lines) to each curve. The slope of each respective tangent is presented in table S1. (**K**) The slope of each tangent in **J** is plotted for the respective samples, showing a gradual solid-to-liquid phase transition as the Ca^{2+} concentration is reduced. (**L**) Time evolution of ±1 defects (purple line) and the spatial correlation length ξ (black line) after EGF stimulation with high calcium concentration ($[Ca^{2+}]$ = 1.12 mM), at time t=0h. (**M**) Time evolution of ±1 defects (blue line) and the spatial correlation length ξ (black line) after EGF stimulation with low calcium concentration ($[Ca^{2+}]$ = 0.07 mM), at time t=0h.

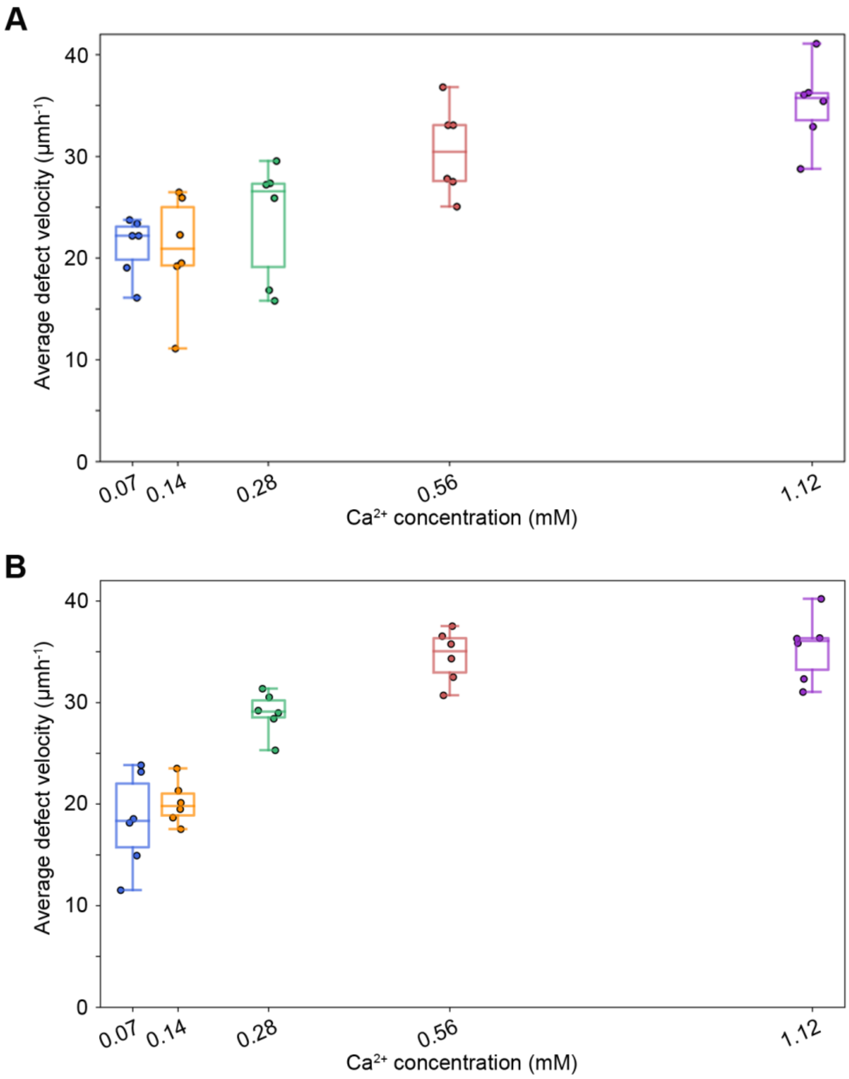

Fig. S6. Analysis of ±1 defect velocity

(**A**-**B**) Box plot depicting the average velocity for (**A**) -1 and (**B**) +1 defects in monolayers treated with EGF and different Ca^{2+} concentrations. Average values are shown for n=6 separate monolayers per sample. The whiskers include minimum and maximum values.

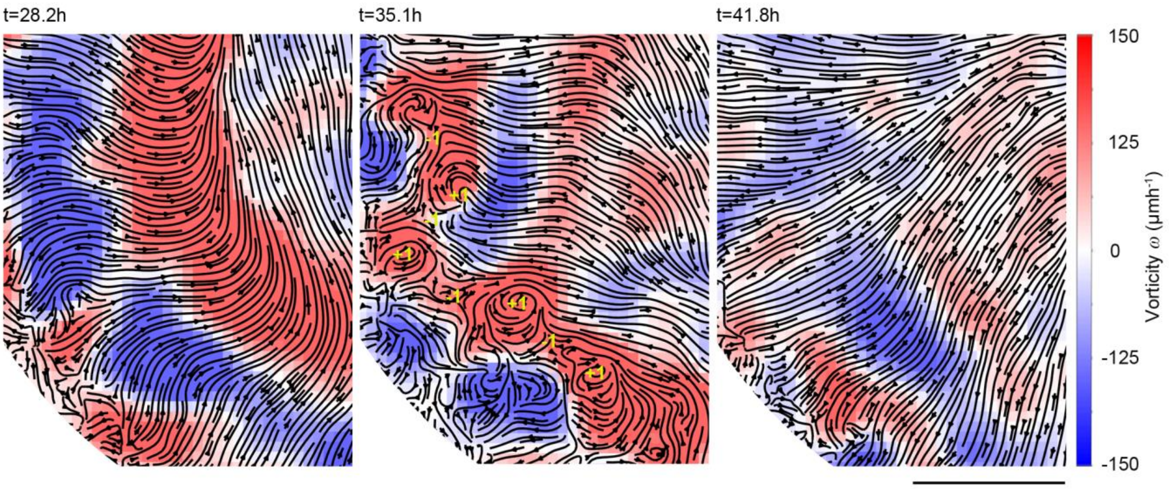

Fig. S7. Dynamic reversal of motility direction along defect arrays in a monolayer
Snapshots of the monolayer velocity field showing the emergence and disappearance of interconnected arrays of ±1 defects along a region of uniform spin orientation. A streamline plot with the vorticity field (color bar) is shown. Scale bar, 1 mm.

Tables

Table S1. Linear regression analysis on average squared distance (μm^2) over time for pairs of migrating cells.

**Stimulation**	**Slope of tangent** (fig. S5, J and K)
EGF + 0.07 mM [Ca^{2+}]	4334.608
EGF + 0.14 mM [Ca^{2+}]	1473.798
EGF + 0.28 mM [Ca^{2+}]	1089.684
EGF + 0.56 mM [Ca^{2+}]	326.371
EGF + 1.12 mM [Ca^{2+}]	157.492
FBS	-25.386

Movie captions

Movie S1. ±1 Defect dynamics in serum-activated quiescent monolayers
Time series showing detection and mapping of ±1 defects. Nuclei are labeled with mCherry-tagged Histone H2B (green), and the normalized vector field shows the direction of cell migration. Circles and diamonds indicate computer-based detection of +1 and -1 defects, respectively. Scale bar, 1000 µm. Microscopy settings: 4x air objective, a time interval of 16 min and a total imaging period of 50 h. The movie displays defect dynamics between 2.5 to 20 h after stimulation.

Movie S2. Defect with charge -1
Time series showing a stable -1 defect. The defect core is detected by a computer algorithm and labeled as an orange diamond. The normalized velocity field is visualized with red arrows superimposed on phase contrast images. Scale bar, 100 µm. Microscopy settings: 20x air objective, a time interval of 4 min and a total imaging period of 48 h. The movie displays approximately 4 h of imaging.

Movie S3. Defect with charge +1
Time series showing a stable +1 defect. The defect core is detected by a computer algorithm and labeled as a purple circle. The normalized velocity field is visualized with red arrows superimposed on phase contrast images. Scale bar, 100 µm. Microscopy settings: 20x air objective, a time interval of 4 min and a total imaging period of 48 h. The movie displays approximately 3 h of imaging.

Movie S4. ±1 defect annihilation
Time series showing mutual attraction and annihilation of a ±1 defect pair. The normalized velocity field is visualized with red arrows superimposed on phase contrast images. Purple circle and orange diamond indicate +1 and -1 defect cores, respectively. Scale bar, 100 µm. Microscopy settings: 20x air objective, a time interval of 4 min and a total imaging period of 48 h. The movie displays approximately 6 h of imaging.

Movie S5. Formation of defect pairs
Time series showing spontaneous formation of a ±1 defect pair. The normalized velocity field is visualized with red arrows superimposed on phase contrast images. Purple circle and orange diamond indicate +1 and -1 defect cores, respectively. Scale bar, 100 µm. Microscopy settings: 20x air objective, a time interval of 4 min and a total imaging period of 48 h. The movie displays approximately 5.5 h of imaging.

Movie S6. ±1 Defect dynamics in monolayers cultured in squared shaped confinement
Time series showing detection and mapping of ±1 defects. Nuclei are labeled with mCherry-tagged Histone H2B (green), and the normalized vector field shows the direction of cell migration. Circles and diamonds indicate computer-based detection of +1 and -1 defects, respectively. Scale bar, 1000 µm. Microscopy settings: 4x air objective, a time interval of 32 min and a total imaging period of 30 h. The movie displays defect dynamics between 2.3 to 18.3 h after stimulation.

Movie S7. ±1 Defect dynamics in the vicinity of a scratch wound
Time series showing detection and mapping of ±1 defects. Nuclei are labeled with mCherry-tagged Histone H2B (green), and the normalized vector field shows the direction of cell migration. Circles and diamonds indicate computer-based detection of +1 and -1 defects, respectively. Scale bar, 500 µm. Microscopy settings: 10x air objective, a time interval of 32 min and a total imaging period of 50 h. The movie displays defect dynamics between 3.4 to 50.7 h after stimulation.

Movie S8. Numerical simulations of monolayer coarsening dynamics
Numerical simulations showing defect annihilation and coarsening of the velocity field over time. Circles and diamonds indicate computer-based detection of defects with charge +1 and -1, respectively. The field contains N=100000 particles.

Movie S9. Visualization of relative cell movement
Time series visualizing relative cell movement in cell sheets stimulated with EGF and low calcium concentration ([Ca^{2+}] = 0.07 mM), EGF and high calcium concentration ([Ca^{2+}] = 1.12 mM), and FBS. Cell trajectories relative to an anchor cell (highlighted by a white square), which is kept at the image center by computer-based registration, are shown. Scale bar, 50 µm. Microscopy settings: 4x air objective, a time interval of 4 min and a total imaging period of 30 to 50 h. The movie displays cellular movements between 20 to 23 h after stimulation.

Movie S10. Defect dynamics in the presence of high [Ca^{2+}]
Time series showing dynamics of ±1 defect pairs in keratinocyte monolayers stimulated with EGF and 1.12 mM Ca^{2+}. The movie shows the velocity field (streamline plot), and automatic labeling of +1 and -1 defects (purple circles and orange diamonds, respectively) superimposed on mCherry-labeled cell nuclei (green). Arrows indicate cell speed. Microscopic settings: 4x air objective, a time interval of 4 min and a total imaging period of 50 h. The movie displays 36 h of imaging with a frame rate of 8 min.

Movie S11. Defect dynamics in the presence of low [Ca^{2+}]

Time series showing dynamics of ±1 defect pairs in keratinocyte monolayers stimulated with EGF and 0.07 mM Ca^{2+}. The movie shows the velocity field (streamline plot), and automatic labeling of +1 and -1 defects (purple circles and orange diamonds, respectively) superimposed on mCherry-labeled cell nuclei (green). Arrows indicate cell speed. Microscopic settings: 4x air objective, a time interval of 4 min and a total imaging period of 50 h. The movie displays 36 h of imaging with a frame rate of 8 min.

Movie S12. Defect dynamics in FBS-stimulated HaCaT WT cells

Time series showing dynamics of ±1 defect pairs in keratinocyte monolayers stimulated with FBS. The movie shows the velocity field (streamline plot), and automatic labeling of +1 and -1 defects (purple circles and orange diamonds, respectively) superimposed on mCherry-labeled cell nuclei (green). Arrows indicate cell speed. Microscopic settings: 4x air objective, a time interval of 8 min and a total imaging period of 30 h. The movie displays 14 h of imaging (from 7 to 21 h after FBS-stimulation) with a frame rate of 8 min.

Movie S13. Defect dynamics in α–Catenin depleted cells

Time series showing dynamics of ±1 defect pairs in α-Catenin-depleted keratinocyte monolayers stimulated with FBS. The movie shows the velocity field (streamline plot), and automatic labeling of +1 and -1 defects (purple circles and orange diamonds, respectively) superimposed on mCherry-labeled cell nuclei (green). Arrows indicate cell speed. Microscopic settings: 4x air objective, a time interval of 8 min and a total imaging period of 30 h. The movie displays 19.5 h of imaging (from 1.3 to 21 h after FBS-stimulation) with a frame rate of 8 min.

Movie S14. Defect annihilations relative to +1 defect spin orientation

Time series demonstrating annihilation of ±1 defects relative to the vorticity field for keratinocyte monolayers stimulated with EGF and 1.12 mM Ca^{2+}. The movie shows streamline plots together with the vorticity field. Red and blue colors represent counterclockwise and clockwise spin orientation, respectively. Circles and diamonds indicate computer-based detection of +1 and -1 defects, respectively. Annihilation events between +1 and -1 defects are indicated by yellow circles. Microscopic settings: 4x air objective, a time interval of 4 min and a total imaging period of 50 h. The movie displays annihilation events between 4.0 and 16.0 h after stimulation.

Movie S15. Polar ordering mediated by neighboring defect pairs with the same spin orientation

Time series displaying the process of annihilation between two neighboring defect pairs with the same spin orientation. The upper panel shows a streamline plot and the vorticity field (color bar). The lower panel displays the velocity field (red arrows) superimposed on mCherry-labeled cell nuclei (shown in gray). Circles and diamonds indicate computer-based detection of +1 and -1 defects, respectively. Microscopic settings: 20x air objective, a time interval of 4 min and a total imaging period of 48 h. The movie displays annihilation events between 13 and 20 h after stimulation with EGF in the presence of 1.12 mM Ca^{2+}.

Movie S16. Polar ordering mediated by neighboring defect pairs with opposite spin orientation

Time series displaying the process of annihilation between two neighboring defect pairs with opposite spin orientation. The upper panel shows a streamline plot and the vorticity field (color bar). The lower panel displays the velocity field (red arrows) superimposed on mCherry-labeled cell nuclei (shown in gray). Circles and diamonds indicate computer-based detection of +1 and -1 defects, respectively. Microscopic settings: 20x air objective, a time interval of 4 min and a total imaging period of 48 h. The movie displays annihilation events between 18 and 25 h after stimulation with EGF in the presence of 1.12 mM Ca^{2+}.

Movie S17. Topology-mediated polarity flipping

Time series demonstrating the mechanism responsible for topology-dependent polarity flipping. The movie shows the velocity field (red arrows) superimposed on mCherry-labeled cell nuclei (shown in gray). Circles and diamonds indicate computer-based detection of +1 and -1 defects, respectively. Scale bar, 500 µm. Microscopic settings: 20x air objective, a time interval of 16 min and a total imaging period of 48 h. The movie displays approximately 9 h of imaging representing time frames t=18.8h to t=27.8h after stimulation with FBS.
